# Are Automated and Visual Greulich and Pyle-Based Methods Applicable to Caucasian European Children With a Moroccan Ethnic Origin When Assessing Bone Age?

**DOI:** 10.7759/cureus.13478

**Published:** 2021-02-21

**Authors:** Grammatina Boitsios, Alessandro De Leucio, Marco Preziosi, Laurence Seidel, Maria P Aparisi Gómez, Paolo Simoni

**Affiliations:** 1 Radiology, Queen Fabiola Children's University Hospital, Brussels, BEL; 2 Biostatistics, University Hospital (CHU) of Liège, Liège, BEL; 3 Radiology, Auckland City Hospital, Auckland, NZL; 4 Radiology, Vithas Hospital October 9, Valencia, ESP

**Keywords:** age determination by skeleton/methods, computer-assisted diagnosis, children/adolescents, ethnic groups, humans

## Abstract

Introduction

To test the accuracy of the visual and automated bone age assessment base on the Greulich and Pyle (GP) method in healthy Caucasian European children with a Moroccan ethnic origin.

Material and methods

Moroccan Caucasian (MC) children were retrospectively and consecutively enrolled along with age- and sex-matched control group (CG) of European Caucasian (EC) children enrolled from the general population. The two groups included 423 children aged from 2 to 15 years with a normal left-hand radiograph performed to rule out a trauma between March 2008 and December 2017. One radiologist, blinded to the BoneXpert^® ^(Visiana, Holte, Denmark) estimates, visually reviewed the radiographs using the GP atlas. The BoneXpert^® ^automatically analysed all 423 radiographs. The intraclass correlation coefficient (ICC), linear regression and Bland-Altman plots were performed to describe the agreement between each method and the chronological age (CA) and the agreement between the two methods.

Results

Visual bone age assessment was related to the CA in both girls (MC ICC 0.97; EC ICC 0.97) and boys (MC ICC 0.95; EC ICC 0.96). Automated bone age assessment was related to the CA in both girls (MC ICC 0.97; EC ICC 0.96) and boys (MC ICC 0.88; EC ICC 0.96). Bland-Altman plots showed an excellent agreement between the two methods in both sexes and ethnicities before puberty especially in Moroccan boys.

Conclusion

Visual and automatic bone age assessment based on the GP method, previously validated in the general population of Caucasian European children, can be confidently used in healthy Caucasian European children with a Moroccan ethnic origin.

## Introduction

The Greulich and Pyle (GP) atlas [1] is the most widely used method to determine bone age in children and adolescents [2]. The GP atlas was introduced in 1959 and developed using a sample of 1,000 left-hand radiographs of healthy middle-class American children of both sexes from 0 to 18 years [3]. The GP atlas was implemented on a particular population, living in a specific environment and socioeconomic conditions [1]. Bone age is determined comparing the hand and wrist radiograph to the GP atlas. The GP atlas has a rapid learning-curve and is particularly suited for less-experienced readers.

Furthermore, the GP atlas has limited accuracy, reproducibility, and inter-observer agreement for patients’ follow-up with growth disturbances [4,5]. Indeed, the standard deviation of bone age estimation using the GP atlas is estimated as 15 months [6].

Automated methods estimating bone age were introduced in the early 2000s to improve the throughput, the accuracy and the reproducibility of bone age assessment [7]. BoneXpert® (Visiana, Holte, Denmark) is one of the validated software to estimate the bone age. BoneXpert® performs automatic modelling of metacarpal and carpal bones of the hand and wrist radiograph [8]. BoneXpert® automatically models fifteen different anatomical regions of the hand and wrist. An iterative analysis provides a bone age estimation according to the GP atlas. The details of this atlas-based iterative processing are described elsewhere [8]. BoneXpert® provides continuous bone age estimates in years and their decimal. A standardised deviation score (SDS) for each 0.5-1 year of interval of age based on the software atlas and iterative process is also provided [7].

BoneXpert® was proven accurate in clinical settings in girls between 2 and 15 years and boys between 2.5 and 17 years [9]. BoneXpert® was validated in a large cohort of healthy children of different ethnic groups, including European Caucasian, American, Chinese and Hispanic [10]. These studies showed how different skeletal maturation can come across different ethnic groups, especially when assessing girls and boys separately [10].

As an example, in the white American population on which the GP atlas was based, it has been highlighted that skeletal maturation has been increasing by about 0.22 to 0.66 years per decade [11].

A recent meta-analysis pointed out that the GP atlas should be used with caution in Asian males and African females [12]. Moreover, previous investigations [10] highlighted the importance to tailor the automatic software for specific ethnic groups to have a more accurate bone age estimation. Even if in the United Kingdom and in the Netherlands, there is a high rate of children with ancestors of different origins in their one or two previous generations, the validation of the BoneXpert® software in different ethnic groups was carried out without taking into account the ethnic origin. This lack of proof relying on the specific evaluation of other ethnic groups could hide different bone maturation patterns demonstrated amongst different ethnicities [13]. As in some European countries like ours, there is a large community of children with a Moroccan ethnic background ancestor in the last one or two generations (as high as approximately 30% of patients referred to our institution). Even if the Moroccans are genetically considered Caucasian descendants, this population was not specifically studied. Therefore, the visual and automatic software was used without a formal confirmation of its validity as other Caucasian ethnic groups such as Dutch [9] and United Kingdom children [14]. For those children, BoneXpert® was not validated as for other populations, even if the automatic assessment of bone age is extensively used in clinical practice.

The aim of this investigation was to test the robustness of the BoneXpert® analysis and the visual assessment based on the GP methods in healthy Caucasian European children with a Moroccan ethnic origin compared to the Caucasian European children.

## Materials and methods

Population

This monocentric retrospective study was approved by the local Ethical Committee (n° CEH N82/17). Clinical records for each patient were available in the institutional Radiology Information System (RIS). No consent was requested from the parents and legal tutors of the children because data were anonymised. Children who underwent a radiograph of their left hands to rule out trauma or musculoskeletal abnormalities between March 2008 and December 2017 in our Picture Archiving and Communicating System (PACS, AGFA version 6.5.3.1509, 2013) were consecutively included in this retrospective study.

Inclusion Criteria

- Children and adolescents aged between 2 and 15 years for the girls and 3 to 15 years for the boys according to the range of ages validated for the automated software [9]. Ages 0 to 2 years old were excluded because of a limited number of cases.

- Normal left hand and wrist radiograph without osteoarticular abnormalities 

Exclusion Criteria

- Known metabolic diseases

- Radiological sequelae of a left hand and wrist trauma

- Previous left-hand surgery with prosthetic devices

- Inherited and developmental bone growth disturbances 

- Myopathies 

- Oncologic diseases.

These exclusion criteria were applied by checking the clinical records of enrolled children on the RIS system of our institution.

Children were assigned in two different groups: 

- The first group consisted of 211 Caucasian European children of Moroccan ethnic origin. Noticeably, children whose Moroccan ethnic origin was uncertain were automatically enrolled in the general control population of Caucasian children. 

- A sex- and age-matched control group of 212 Caucasian European outpatients were selected, that is, from the general population of our institution. 

Based on their demographic features, children were assigned to a one-year and sex-class of age. For each sex- and age-group (e.g., female, 11 years old), a maximum number of 10 patients were enrolled. After the database was built, the identity of the patients and legal tutors were anonymised except for sex and age. The first investigator was the guarantor of the data anonymisation and the legal privacy requirements. 

Images acquisition

In our institution, dorso-palmar radiographs of left hand to rule out a musculoskeletal trauma are acquired on a Siemens Multix TOP radiograph table equipped with an Oldelft Computed Radiography (CR) detector. The standardised protocol is the following: distance from tube: 1 meter; tube tension 48 kV; filter 0.1-0.3 mm Cu. The mAs were automatically determined by the automatic exposure device (AED) system. The oblique view of the hand was used to rule out the trauma but not taken into account in the present study for the bone age estimation. 

Images analysis

Visual Assessment by a Blinded Radiologist Using the GP Atlas

Each image was anonymised for the name and stored on the PACS for the blinded visual analysis by one single reader 10-year board-certified radiologist (GB). Noticeably, in the visual examination, the radiologist took into account all the bones including the carpal bones and the narrative description of the bone shape features of each age provided in the page annexed to each image of the physical book version of the GP atlas. The images were examined on a Barco medical display (MDCC-6230). The order of the radiograph review was randomised using the www.random.org website between 12 and 16 February 2018.

Automatic Analysis by BoneXpert®

BoneXpert® software (Visiana, Holte, Denmark, version 2.4.5.1) analysed all the radiographs. The native images, along with the processed images, were stocked into the PACS. The GP bone age and the SDS provided by the BoneXpert® were noted on an Excel worksheet.

**Figure 1:**
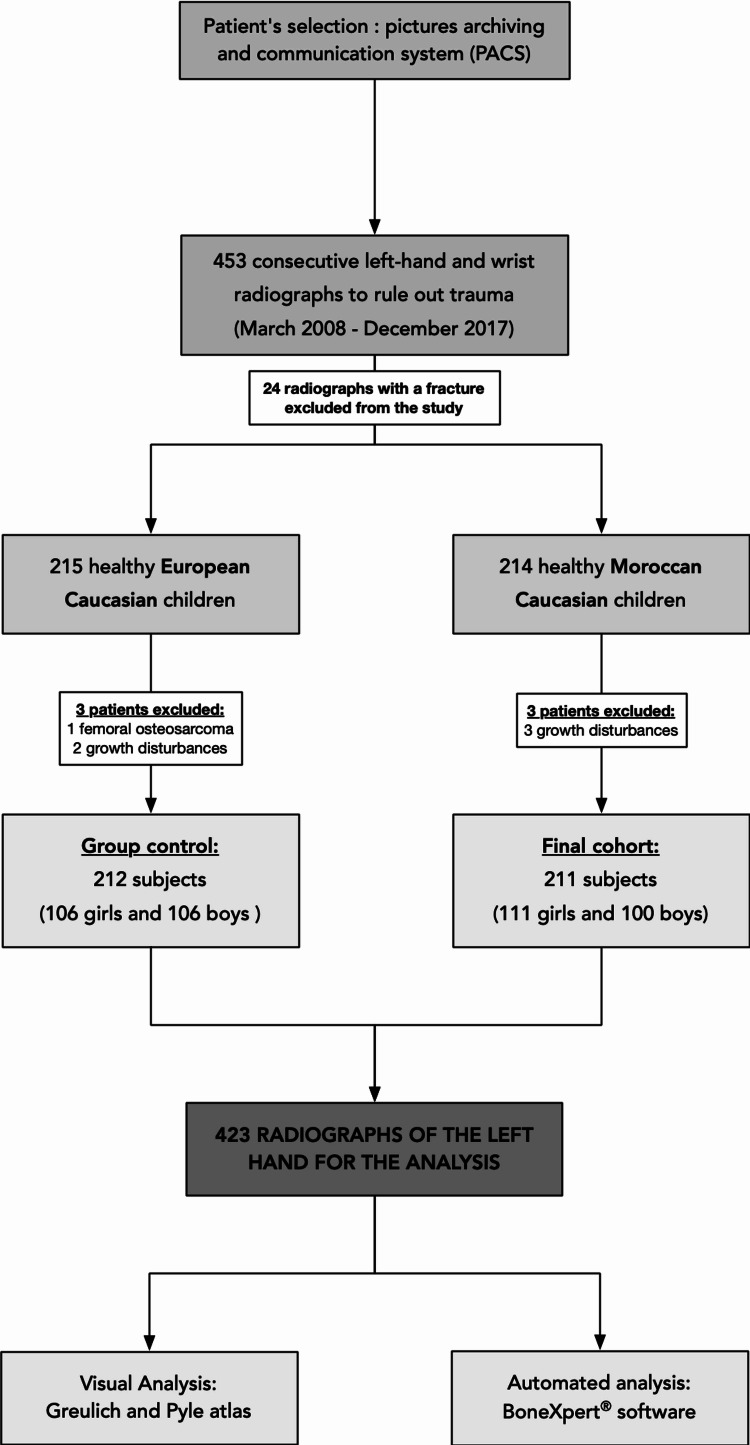
Study design.

The study design is summarised in Figure 1.

Statistical Analysis

Results were expressed as mean and standard deviation (SD). Frequency tables were used for categorical findings. The intraclass correlation coefficient (ICC) with 95% lower confidence limit (LC95%), linear regression and Bland-Altman plots were used to assess the consistency of bone age with the known chronological age as well as for the comparison of the two methods using the IBM SPSS Statistics (version 27). Results were considered significant at the 5% significant level (p<0.05). Statistical analysis was carried out by a professional academic biostatistician (LS).

## Results

In total, 453 radiographs were eligible to be enrolled in the study, of which 24 radiographs were excluded because the radiograph showed a traumatic lesion. The results were composed of 429 radiographs of 215 European Caucasian and 214 Moroccan Caucasian children (Figure 1). Of these, three radiographs in the control group were excluded (one for osteosarcoma and two for growth disturbances) and three radiographs were excluded from the Moroccan Caucasian group because of growth disturbances. The final cohort was constituted of 211 Moroccan children (111 girls and 100 boys) and 212 European children (106 girls and 106 boys). The descriptive statistics are shown in Table 1. 

**Table 1:** Descriptive statistics of the cohort.

		All		Moroccan girls		European girls		Moroccan boys		European boys	
Variable	Categories	N	Number (%)	N	Number (%)	N	Number (%)	N	Number (%)	N	Number (%)
Sex		423		111		106		100		106	
	Female		217 (51.3)		111 (100.0)		106 (100.0)		0 (0.0)		0 (0.0)
	Male		206 (48.7)		0 (0.0)		0 (0.0)		100 (100.0)		106 (100.0)
Ethnicity		423		111		106		100		106	
	Moroccan		212 (50.1)		0 (0.0)		106 (100.0)		0 (0.0)		106 (100.0)
	European		211 (49.9)		111 (100.0)		0 (0.0)		100 (100.0)		0 (0.0)
Age categories (years)		423		111		106		100		106	
	2		14 (3.3)		10 (9.0)		4 (3.8)		0 (0.0)		0 (0.0)
	3		37 (8.7)		10 (9.0)		10 (9.4)		7 (7.0)		10 (9.4)
	4		30 (7.1)		6 (5.4)		5 (4.7)		10 (10.0)		9 (8.5)
	5		33 (7.8)		9 (8.1)		9 (8.5)		8 (8.0)		7 (6.6)
	6		27 (6.4)		7 (6.3)		5 (4.7)		8 (8.0)		7 (6.6)
	7		39 (9.2)		10 (9.0)		10 (9.4)		9 (9.0)		10 (9.4)
	8		31 (7.3)		8 (7.2)		9 (8.5)		7 (7.0)		7 (6.6)
	9		31 (7.3)		10 (9.0)		9 (8.5)		5 (5.0)		7 (6.6)
	10		33 (7.8)		10 (9.0)		9 (8.5)		7 (7.0)		7 (6.6)
	11		31 (7.3)		8 (7.2)		7 (6.6)		8 (8.0)		8 (7.5)
	12		30 (7.1)		6 (5.4)		8 (7.5)		8 (8.0)		8 (7.5)
	13		30 (7.1)		7 (6.3)		8 (7.5)		7 (7.0)		8 (7.5)
	14		30 (7.1)		7 (6.3)		7 (6.6)		7 (7.0)		9 (8.5)
	15		27 (6.4)		3 (2.7)		6 (5.7)		9 (9.0)		9 (8.5)

Caucasian Moroccan children

In the Moroccan Caucasian group, the correlation between the automated bone age estimations and the chronological age showed a high correlation in determining the bone age in comparison to the chronological age. As well as the automated method, the visual analysis showed a high correlation in the estimation of the bone age. 

*Automated (BoneXpert®) Bone Age Assessment* 

**Figure 2:**
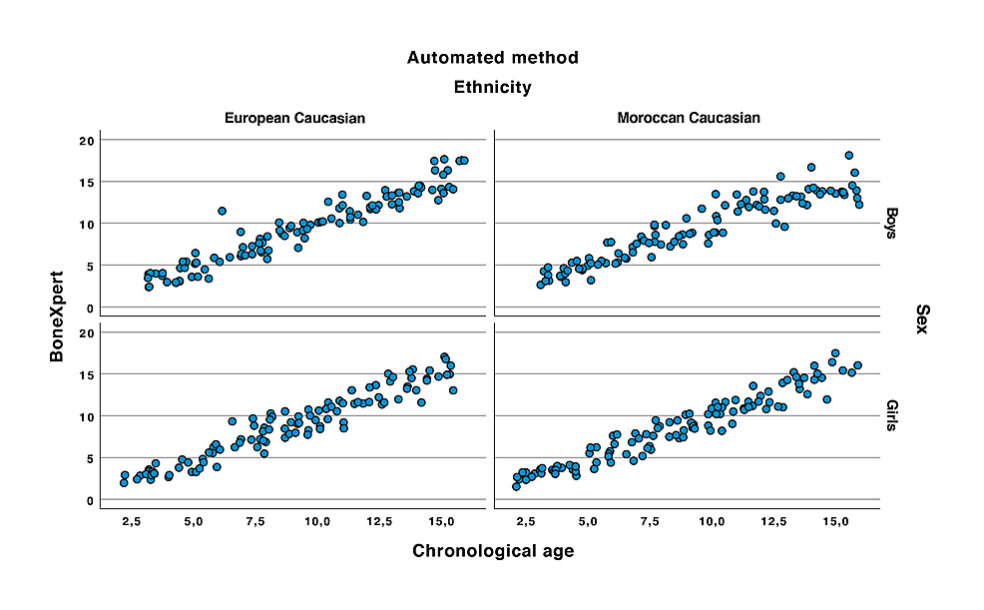
Correlation between the automated method and the chronological age.

**Figure 3:**
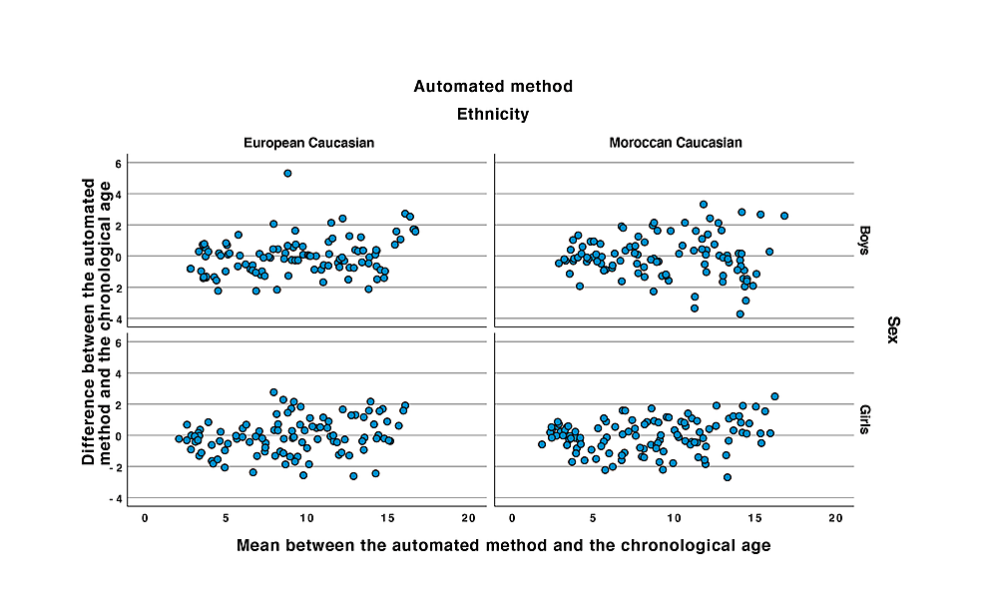
Bland and Altman plots of the automated method plotted to the chronological age.

Automated bone age accuracy in Caucasian Moroccan girls:

Linear regression analysis showed that the automated bone age assessment was accurate to determine bone age in healthy Caucasian European girls with Moroccan ethnic origin (r=0.966 and p<0.0001) (see Figure 2). The ICC was 0.97 (lower confidence LC95% = 0.95). The mean of the differences in the Bland and Altman plot was equal to -0.05 (see Figure 3).

Automated bone age accuracy in Caucasian Moroccan boys:

Linear regression analysis showed that the automated bone age was accurate to determine bone age in healthy Caucasian European boys with Moroccan ethnic origin (r=0.939 et p<0.0001) (see Figure 2). The ICC was 0.88 (LC95% = 0.92). The mean of the differences in the Bland and Altman plot equal to -0.06 (see Figure 3).

Visual (GP Atlas) Bone Age Assessment

**Figure 4:**
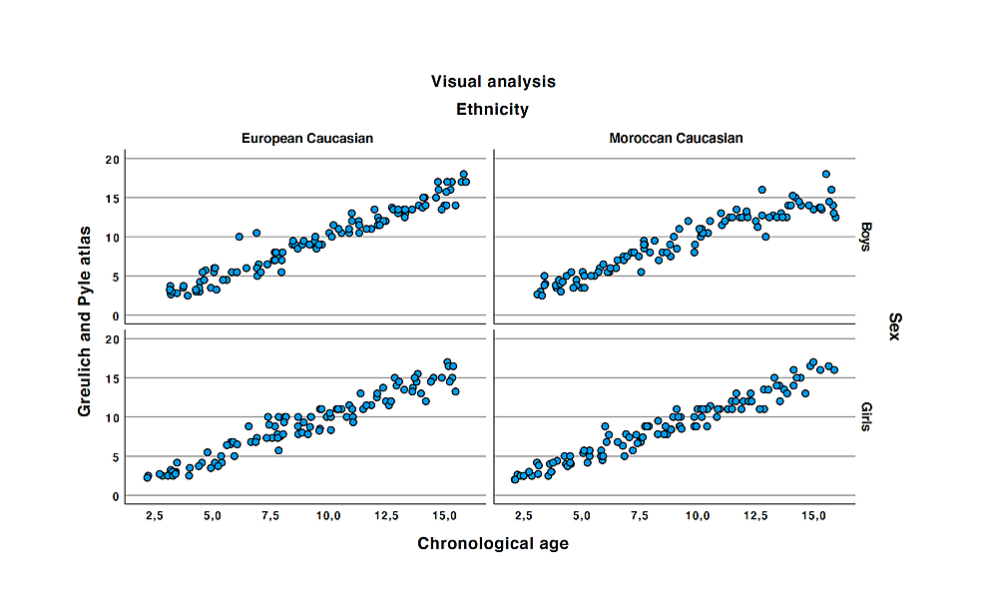
Correlation between the Greulich and Pyle and the chronological age.

**Figure 5:**
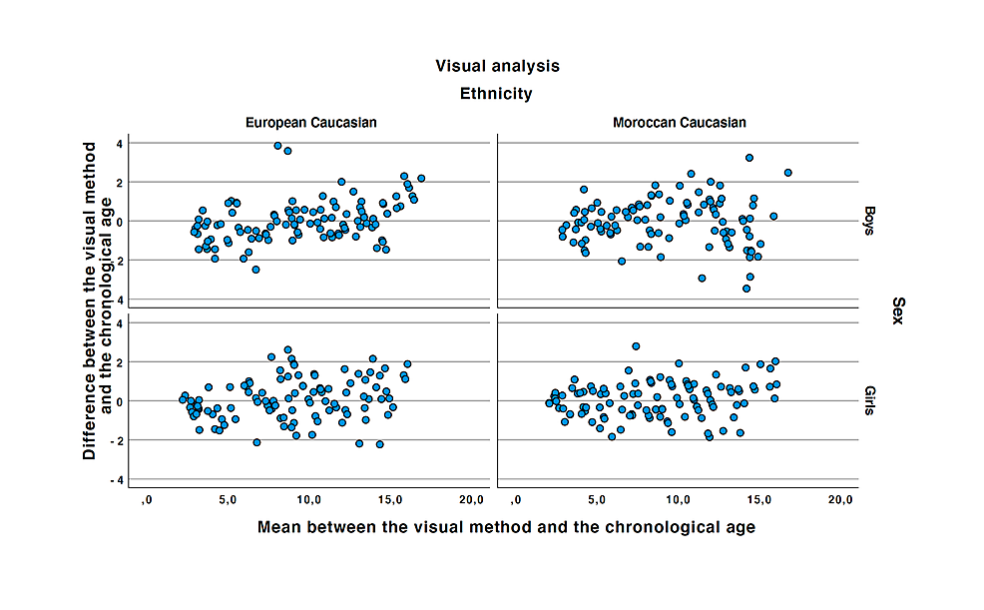
Bland and Altman plots of the Greulich and Pyle atlas plotted to the chronological age.

Visual analysis accuracy in Caucasian Moroccan girls:

Linear regression analysis showed that the GP atlas was accurate to determine bone age in healthy Caucasian European girls with a Moroccan ethnic origin (r=0.973 and p<0.001) (see Figure 4). The ICC was 0.97 (LC95% = 0.96). The mean of the differences in the Bland and Altman plot equal to 0.08 (see Figure 5).

Visual analysis accuracy in Caucasian Moroccan boys:

Linear regression analysis showed that the GP atlas was accurate to determine bone age in healthy Caucasian European boys with Moroccan ethnic origin (r=0.954 and p<0.001) (see Figure 4). The ICC was 0.95 (LC95% = 0.94). The mean of the differences in the Bland and Altman plot equal to -0.04 (see Figure 5).

Caucasian European children

In the control group, the correlation between the automated bone age estimation and the chronological age showed a high correlation in determining the bone age compared to the chronological age. The bone age estimation's visual analysis also showed a high correlation with the chronological age.

*Automated (BoneXpert®) Bone Age Assessment* 

Automated BoneXpert® bone age accuracy in Caucasian European girls:

Linear regression analysis showed that the automated bone age was accurate to determine bone age in healthy Caucasian North European girls (r=0.957 and p<0.0001) (see Figure 2). The ICC was 0.96 (LC95% = 0.94). The mean of the differences in the Bland and Altman plot equal to -0.07 (see Figure 3).

Automated BoneXpert® bone age accuracy in Caucasian European boys:

Linear regression analysis showed that the automated software was accurate to determine bone age in healthy Caucasian North European boys (r=0.957 et p<0.0001) (see Figure 2). The ICC was 0.96 (LC95% = 0.94). The mean of the differences in the Bland and Altman plot equal to -0.02 (see Figure 3).

*Visual (GP Atlas) Bone Age Assessment* 

GP atlas accuracy in Caucasian European girls:

Linear regression analysis showed that the GP atlas was accurate to determine bone age in healthy Caucasian North European girls (r=0.965 and p<0.001) (see Table 4). The ICC was 0.97 (LC95% = 0.95). The mean of the differences in the Bland and Altman plot equal to 0.11 (see Figure 5).

GP atlas accuracy in Caucasian European boys:

Linear regression analysis showed that the GP atlas was accurate to determine bone age in healthy Caucasian European boys (r=0.967 and p<0.0001). (see Figure 4). The ICC was 0.96 (LC95% = 0.94). The mean of the differences in the Bland and Altman plot equal to -0.02 (see Figure 5).

Comparison between the two methods

**Figure 6:**
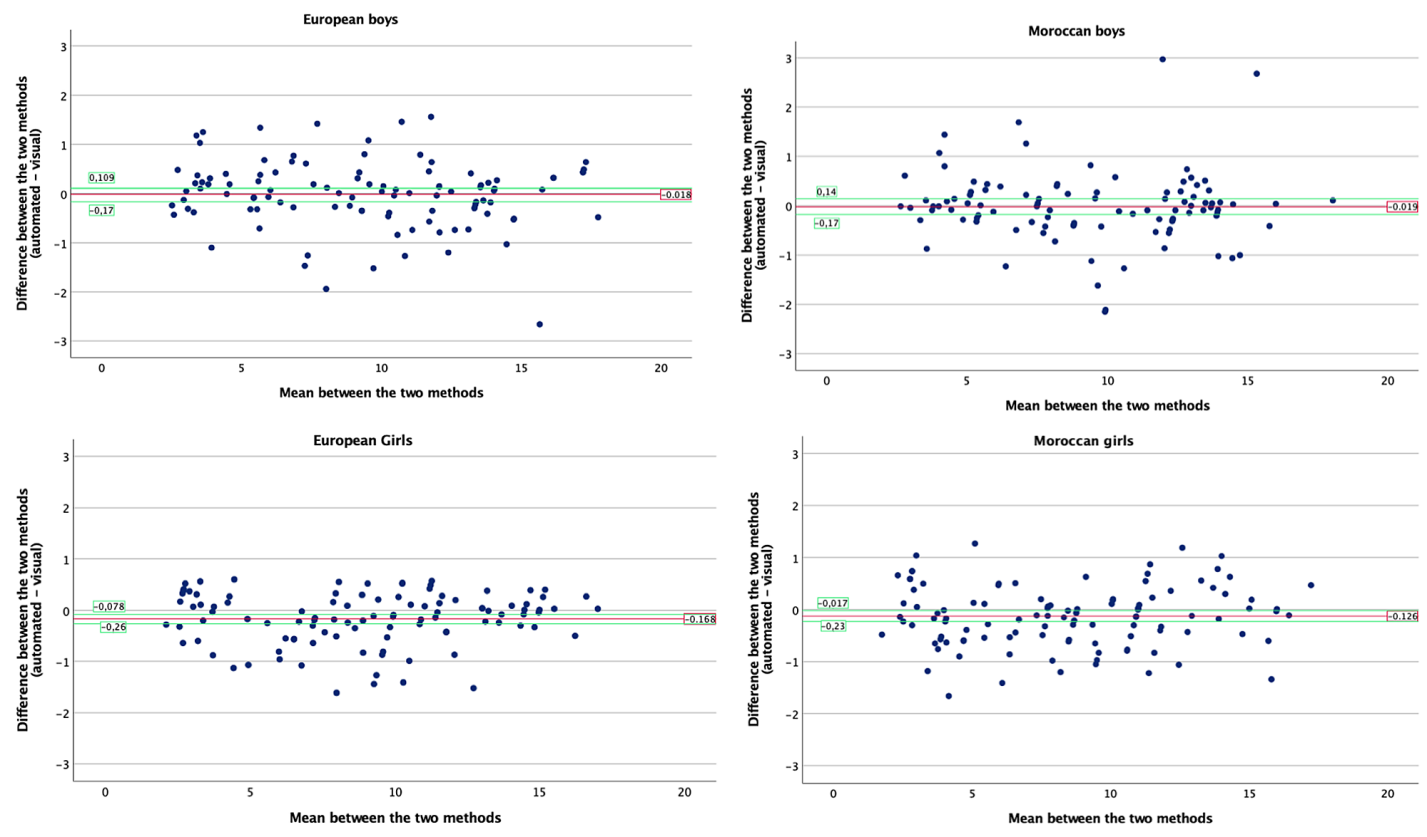
Bland and Altman plots of the mean of the methods plotted to the difference between the two methods (*bootstrap analysis was performed to establish the 95% IC; native values were normally distributed). IC: interval of confidence.

Bland and Altman plots were performed to describe the agreement between the two methods and are depicted in Figure 6. As our data were not normally distributed, a bootstrapping analysis algorithm was performed to determine the upper and lower confidence interval. The four groups' means are almost equal to zero, showing an excellent agreement between the two methods. 

In Moroccan girls, although the distribution of the data around the mean is relatively homogeneous, a concentration over the mean is observed between two and four years of age showing higher difference between the automated than the visual method. Also, after puberty (around the age of 13 years), the distribution of the data is focused above the difference’s mean showing higher values for the automated compared to the visual analysis.

In Moroccan boys, the estimates are closer to the mean between 5 and 10 years and after 12.5 years. In European boys, the variability is higher in comparison to the Moroccan boys. Moreover, after puberty the data seem to be above the mean line suggesting a tendency to overestimation of the automated analysis compared to the visual analysis.

The Bland and Altman plot in the European girls represents a denser distribution around the mean line with a positiveness after puberty.

## Discussion

Paediatric radiologists’ favourite method of assessing skeletal maturation in clinical practice is the GP atlas [2]. When determining bone age in children from one to three years and older than three years, the GP atlas is respectively used in 85.9% and 92.4 % of the cases [2]. The higher the patient's age, the higher the confidence level of the radiologist (up to 48% when assessing children older than three years) [2].

The GP atlas was conceived in a different population in terms of socioeconomic and ethnic parameters. Growth patterns amongst different ethnicities are well known [15]. Along with the automated methods in skeletal maturation assessment (e.g., BoneXpert), the interobserver variability can be overcome.

The present study is the first investigation to assess the validity of the visual and automated bone age assessment in healthy Caucasian European children with a Moroccan ethnic origin. Unlike North America, in Europe, the validity of the GP-based methods for evaluating the bone age with both visual and automatic techniques was performed without considering the high rate of children of different ethnic groups. Previous studies, conducted in the United States, highlighted substantial differences in bone maturation patterns amongst children born and raised in the same country but with a different ethnic origin [16].

The high number of Caucasian European children with a Moroccan ethnic origin living in Europe, which varies in proportion between different countries, requires a specific validation of these tools. Because of the high incidence of Moroccan Caucasian European children in several institutions of French-speaking countries, we endeavour to test the validity of the GP-based visual and automated methods in this particular population. 

Our investigation provides us with interesting findings. 

Firstly, the results of this investigation suggest that both visual and automated method assessing skeletal maturation based on the GP methods are valid tools in healthy Caucasian European children with Moroccan ethnic origin, as confirmed by the ICC analysis. Both methods showed a high concordance in determining the bone age when compared to the chronological age in both sexes and both ethnicities. The yielded values are statically equivalent to those observed in the control population enrolled in European children's general population. Hence, both visual and automatic GP atlas-based methods can be confidently used in Caucasian Moroccan children of both sexes.

Secondly, the comparison of each method to the chronological age shows a higher variability of the method determining the bone age especially after puberty. In Moroccan girls, the variability, for both methods, is constant showing no variability during puberty. In Moroccan boys, and for both methods, the variability is higher after puberty. In Caucasian girls, we observe periods of time where the spread shows higher variability (around 8-10 years and after 13 years). In Caucasian boys, the GP atlas shows an underestimation of the bone age before eight years and an overestimation after 8 years of age. 

Thirdly, and since the data were not normally distributed, in the Bland and Altman plots, we performed a classical bootstrapping algorithm with an iterative 400 bootstrap sample in order to obtain a normalised distribution sample and thus to be able to calculate the confidence intervals for the Bland and Altman plots (see Figure 6). As seen on these Bland and Altman plots, the confidence intervals are indeed close to the mean, showing a high concordance between the two methods. Apart from the general higher overall variability between the chronological and the bone age observed in all groups due to the pubertal spur (after 8 in girls and 9 in boys) reported in previous studies, we observed quite a significant variability in Caucasian Moroccan boys compared to other groups. This finding may suggest specific kinetics of bone age progression in Moroccan Caucasian boys that should be further investigated in a larger population enrolled prospectively and related to anthropometric and clinical features.

The GP atlas was proven unreliable in determining bone age, especially in individuals younger than two years because of the lack of ossification of the carpal bones and the metacarpal epiphysis. Hence, other methods to assess skeletal maturity are proposed in children younger than two years [17]. Further studies should test the accuracy of the automated software in determining bone age in toddler younger than two years of age.

This preliminary study paves the way to validating the visual and automated methods in Caucasian European children with Moroccan ethnic origin suffering from growth disturbances. The visual bone age estimation using the GP atlas can confidently be used when the automated software is unavailable both in healthy North European boys and girls with a Moroccan ethnic origin in clinical settings. 

This study has some limitations:

(a) The 423 healthy subjects were retrospectively enrolled in one single centre.

(b) The patients' selection was based only on demographics information in the RIS-PACS, which could have introduced potential bias in patient's enrolment. 

(c) We considered the ethnicity (Moroccan) as a homogeneous entity as in other studies of validation of the BoneXpert® software among different ethnicities [10]. However, this definition can be somehow inaccurate as the definition used in previous studies such as Afro-American and Hispanic because this population can be made of very different a homogeneous ethnic group. This study focused on a population of subjects from a single country reducing bias.

(d) The radiographs were visually reviewed by only one single-blinded radiologist hence preventing to study the inter-observer agreement in the visual assessment.

(e) The bone mass and other anthropometric indexes were not considered in this study.

Our study opens several perspectives. 

Firstly, automated BoneXpert® software should be further studied to validate this method in clinical settings, including predicting the adult target height. Secondly, on a financial basis, the economic impact of this new automated method should be processed. Thirdly, the validity of the BoneXpert® software should be studied in the same ethnic group suffering from growth disturbances to be extrapolated in other pathologies. 

## Conclusions

In conclusion, our study shows that the GP atlas and the BoneXpert® software are valid tools assessing skeletal maturity in healthy Caucasian Moroccan children and adolescents, as well as in the general population. Attention should be drawn assessing bone age in children during puberty where a more significant variability in bone age determination is observed with both methods, particularly in Moroccan boys. Our results suggest a specific growth pattern in Moroccan Caucasian boys that should be further investigated in a large prospective study.
